# Effect of Zinc Dialkyl Dithiophosphate Replenishment on Tribological Performance of Heavy-Duty Diesel Engine Oil

**DOI:** 10.1007/s11249-022-01565-8

**Published:** 2022-02-07

**Authors:** A. Al Sheikh Omar, F. Motamen Salehi, U. Farooq, A. Neville, A. Morina

**Affiliations:** 1 School of Mechanical Engineering, Institute of Functional Surfaces, University of Leeds, Leeds, UK; 2 Racor Filter Division Europe, Parker Hannifin Manufacturing Ltd, Dewsbury, UK

**Keywords:** Soot, Wear, Additive depletion, Additive replenishment

## Abstract

Soot is the main contamination that affects oil performance and increases the frequency of oil changes in heavy-duty engine oil. Several studies discussed that additive concentration in engine oil can be influenced due to additive depletion over time and additive adsorption on soot particles. To extend oil drain intervals and improve oil performance, filter manufactures explore removing the soot to a certain level and replenishing the consumed additives. Zinc dialkyl dithiophosphate (ZDDP) is one of the most favored antiwear additives that react very rapidly with rubbing surfaces to form tribofilm that reduces wear. In this study, the experimental work aims to investigate the effect of ZDDP replenishment on tribological performance in the existence of soot and after removing soot from heavy-duty used oil. The study reveals that reclaiming the used oil can be achieved by removing the soot to a certain level. The results demonstrate that the reclaimed oil after removing soot is still not as good as the fresh oil. This study proves that additive depletion, additive adsorption on soot, and the decomposition of antiwear additive adversely influence the reclaimed oil performance. However, replenishing the consumed additive by adding a small amount of ZDDP helps to improve the reclaimed oil performance compared to a large amount of ZDDP which is required to re-gain the oil performance in the existence of soot.

## Introduction

Engine manufacturers are growing demands to increase the oil drain interval. The US Energy Information Administration reported [1] that over 1 billion gallons ($\mathrm{454,609}\times {10}^{4}$ L) of lubricants annually are replaced in light vehicles and more than 250 million gallons ($\mathrm{4,546,090}$ L) in trucks. Engine manufactures are under considerable pressure to reduce the effects of disposal oil on the environment and improve profitability by extending the service life of engine oils [1]. Soot is one of the main contamination that influences significantly oil drain interval in diesel engines [2, 3]. The effects of soot on the oil performance have been addressed by several studies; some researches focused on the effect of soot on wear [4–8], other studies concentrated on soot effect on tribofilm and rubbing surfaces [9, 10] and finally, additive adsorption on soot particles has been the topic of interest [3, 11–14]. The presence of soot in diesel engine oil is one of the most common reasons to change engine oil [2, 3, 15]. While removing soot by filters can improve the oil functionality, however, there are still depleted additives that need to be replenished to enhance the oil performance.

Additives in engine oils could be consumed, decomposed, or depleted after being used in the engine causing a decrease in the oil performance [16–18]. Engines manufacturers recommend that engine oil should be changed at regular intervals depending on the operating conditions to keep the additive level up. Replenishing these additives can forestall oil degradation and maintain oil properties and its effectiveness for a longer time [16]. In order to prolong the oil change intervals, it has been proposed to add an excess amount of additive to ensure a sufficient quantity of additive exists at all time. The additives are suggested to be added periodically regardless of oil contaminants or the oil usage history [16]. Replenishing the additives can protect the contacts surfaces from abrasive wear caused by soot [19] and prevent oil oxidation and corrosion [18].

Additive replenishment is an interesting topic to the manufacturing sector since it can potentially extend the lubricant’s life. There are many studies regarding the effect of adding additives on fresh oils to enhance the oil functionality [20–22], while the effect of additives replenishment on used oil or reclaimed oil (the oil after removing/filtering the soot) has not been investigated. However, several patents [23–26] have discussed different additive system suppliers to replenish the consumed additives through controlled injectors or filters. Some patents designed oil filters to release additives using such as colloidal suspensions of PTFE particles with a size less than two microns in filter [26]. Polyolefin container or capsules containing additive is studied to release the additive in flowing oil [25]. Soluble composite in filter material such as polymer matrix is used to release the additive [23, 24]. Other patents suggested mechanical mechanisms that can inject the additive into oil circulation systems [26, 27]. All of these techniques have not taken into account that adding too much additive has a negative effect on tailpipe emissions and the vehicle fuel economy. Accordingly, it is important to have a controlled additive technique to keep the additive concentration within the desirable limits [28].

Zinc dialkyl dithiophosphate (ZDDP) is the most common additive which can control wear and act as oxidation and corrosion inhibitor [29]. ZDDP reacts on rubbing surfaces to form a quite thick tribofilm (up to 200 nm) [30]. The effect of ZDDP concentration on tribofilm formation was investigated by Yin et al. [31]. The results revealed that a low concentration of ZDDP (0.25 wt% and 0.5 wt%) forms short phosphate chains, whereas a high percentage of ZDDP (1% and 2%) leads to the formation of long phosphate chains. Furthermore, Tomala et al. [32] found that the larger percentage of ZDDP causes thicker and larger roughness of tribofilm. Ghanbarzadeh et al. [33] indicated that increasing ZDDP concentration increased the formation and thickness of tribofilm and reduced the wear. The formation of tribofilm derived from added ZDDP has a negative effect on friction causing an increase in friction coefficient. The reason behind this is the higher shear strength of tribofilm [34] due to an effective roughening of rubbing surfaces by the formation of uneven distribution of asperity peaks [30].

In the current study, the ZDDP replenishment process in heavy-duty used oil containing soot and after removing the soot is explored. ZDDP is added to used oil to overcome the negative effects caused by soot particles and the additive depletion in the engine. This study determines the amount of the depleted additive and the level of ZDDP needed to extend the service life of heavy-duty used oil in the existence of soot or after reclaiming the used oil by removing soot.

## Experimental Methodology

### Materials and Method

**Fig. 1:**
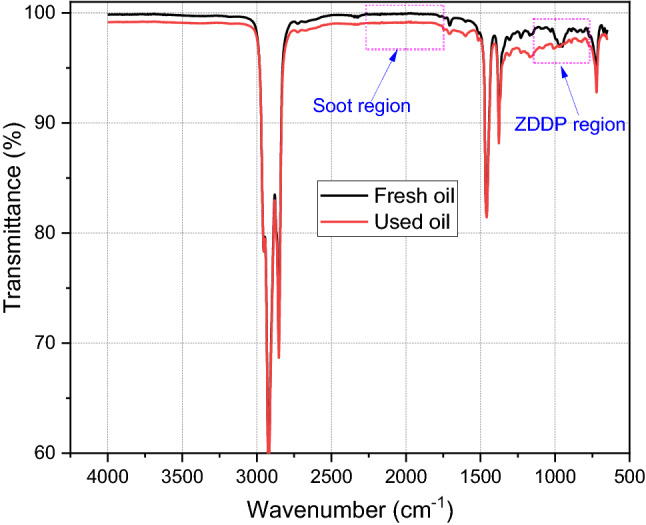
FTIR chemical analysis of used oil compared to fresh oil

**Fig. 2:**
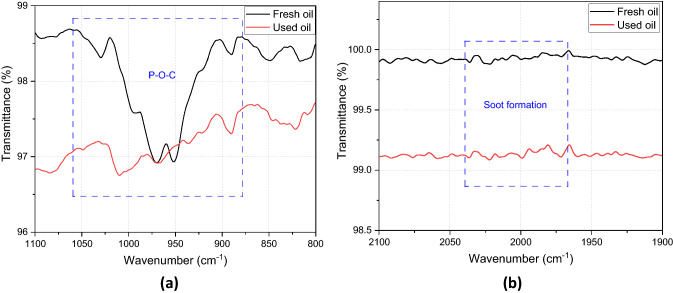
Antiwear additive area at 978 cm^−1^, **b** soot formation area at 2000 cm^−1^

Fully formulated oil (BDS4 oil produced by ExxonMobil) of heavy-duty vehicles with viscosity grade 15W40 was used in this study. The physical and chemical properties of fresh oil are displayed in Table 1. The engine oil after being used in the heavy-duty diesel engine of a truck was drained for further investigations. The drained oil was provided by Parker. The mileage details of drained engine oil are shown in Table 2. Chemical investigation to study the oil degradation was conducted using Fourier-transform infrared spectroscopy (FTIR) manufactured by PerkinElmer. FTIR results demonstrate the change in the oil after being used as shown in Fig. 1. Figure 1 reveals two interesting areas in the used oil spectrum compared to the fresh oil. The 978 cm^−1^ point is correlated to the P–O–C bond which refers to the antiwear additive and the 2000 cm^−1^ point relates to soot level in used oil [35]. Figure 2a shows no peak in used oil at 978 cm^−1^ point that could be due to depletion or decomposition of antiwear additive. Decomposition of antiwear additives at engine conditions was found in previous studies [3, 18]. Figure 2b confirms the existence of soot particles in the used oil as there is a shift in the FTIR spectra at 2000 cm^−1^ according to ASTM D7844 standard [36].

**Table 1:** Physical and chemical properties of fresh oil

Details	Parameters
Density at 15 °C	0.874 g/ml
Kinematic viscosity at 40 °C	113 mm^2^/s
Kinematic viscosity at 100 °C	15 mm^2^/s
Flash point	215 °C
Total base number	10 mgKoH/g
Zn concentration	1306 ppm
P concentration	1158 ppm
S concentration	6366 ppm
Ca concentration (ppm)	1332 ppm
Mg concentration (ppm)	941 ppm

**Table 2:** Used oil details (the oil was changed depending on the mileage in the average between 40,000 and 50,000 km)

Truck oil change	Oil filling	Oil draining
Date	21.06.2019	29.11.2019
Mileage (km)	524,604	563,842

Inductively coupled plasma (ICP) has been used to further investigate the change in additive concentration in the used oil according to ASTM D5185 standard [37]. ICP analysis was conducted at Oil Check Laboratory Services Ltd, UK. KINEXUS rheometer was used to investigate the change in the viscosity at test temperature (100 °C) after being used in the heavy engine. The viscosity of used oil was $0.0097\pm 0.0006$ Pa.s compared to the viscosity of fresh oil $0.0092\pm 0.00007$ Pa.s. The viscosity values show a small decrease in the viscosity of used oil compared to fresh oil. The decrease in the viscosity of used oil is due to loss of Viscosity-Index Improver (VII) at high temperature causing the decrease in the viscosity [38]. Carden et al. [39] showed that a small change in the oil viscosity didn’t influence the wear and friction. However, the ultra-low oil viscosity test revealed an increase in wear.

### Soot Removal by Centrifugation

**Fig. 3:**
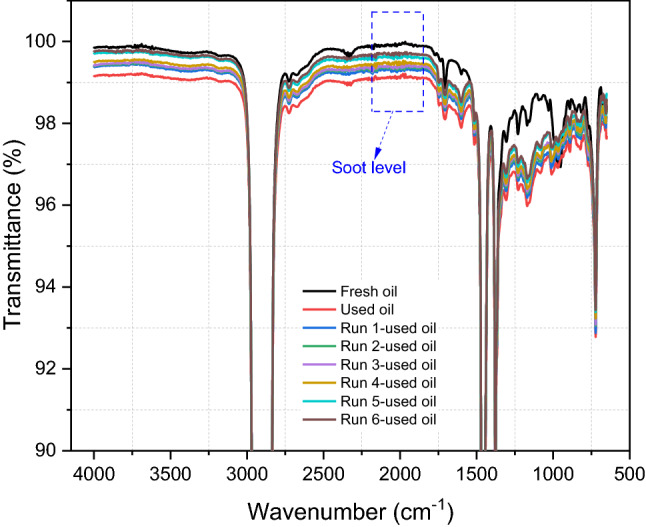
Centrifugal process was repeated 6 times at the same conditions and soot level was measured after every run, the shift at 2000 cm^−1^ point was used to estimate the soot percentage in used oil

**Fig. 4:**
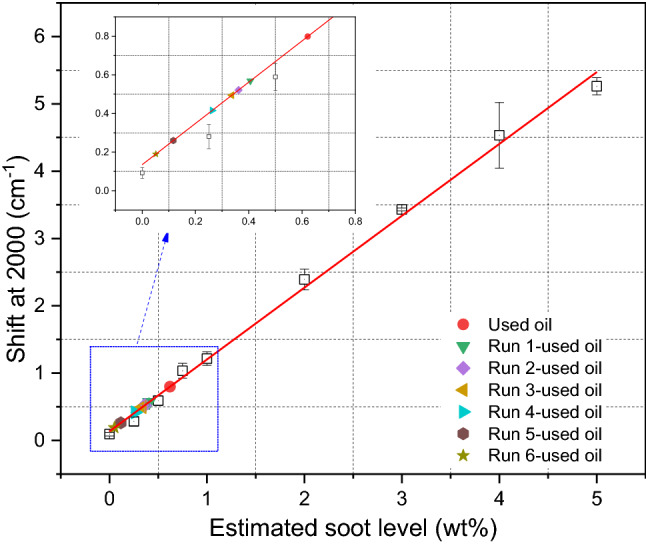
Soot level in used oil was measured at 2000 cm^−1^ point after every centrifuge run by FTIR and plotted on the calibration curve to obtain the soot level in the oil

A centrifuge was used to remove soot particles from the used oil. The centrifuge was conducted at 40 °C with a speed of 12,000 rpm for 2 h. This centrifugation process was repeated six times to ensure that most of the soot has been removed. FTIR was used to measure the soot level in the used oil according to ASTM D7844 standard [36]. Figure 4 shows the calibration curve to estimate soot percentage in used oil plotted between different levels of carbon black (CB) and the shift at the 2000 cm^−1^ point. Fundamentally, soot or carbon black (CB) level in the oil causes the shift at 2000 cm^−1^ point of FTIR spectra and the shift in the spectra is associated with soot or CB level. The CB at varied levels was blended homogeneously in the oil using the hotplate at the temperature of 60 °C and the speed of 500 rpm for 1.5 h. The oil sample containing the known level of CB was measured using FTIR. The calibration curve plotted the relationship between the known CB level in the oil and the shift at 2000 cm^−1^ point of FTIR spectra. The calibration curve was applied in this study to measure the soot level in drained oil after every centrifuge run. Figure 3 shows a decrease in soot percentage in used oil after every run as the FTIR spectra shift up. Drained oil contained 0.62 wt% of soot after being used in the truck. Soot level decreased gradually after every centrifuge run and the soot level after 6-times centrifugation became 0.05 wt% as shown in Fig. 4.

### ZDDP Replenishment Process

ZDDP antiwear additive at different levels (0, 0.75, 1.5, 3, and 5 wt% ZDDP) was used in this study to replenish the consumed additive at both conditions before and after the removal of soot. For all oil samples, a magnetic stirrer was used to mix the ZDDP homogeneously into the oil at 60 °C for 30 min. Tribological test programs after replenishment process were carried out for the used oil with soot and used oil after removing soot (reclaimed oil). In this study, the reclaimed oil referred to the used oil after removing the soot.

### Tribological Test

**Fig. 5:**
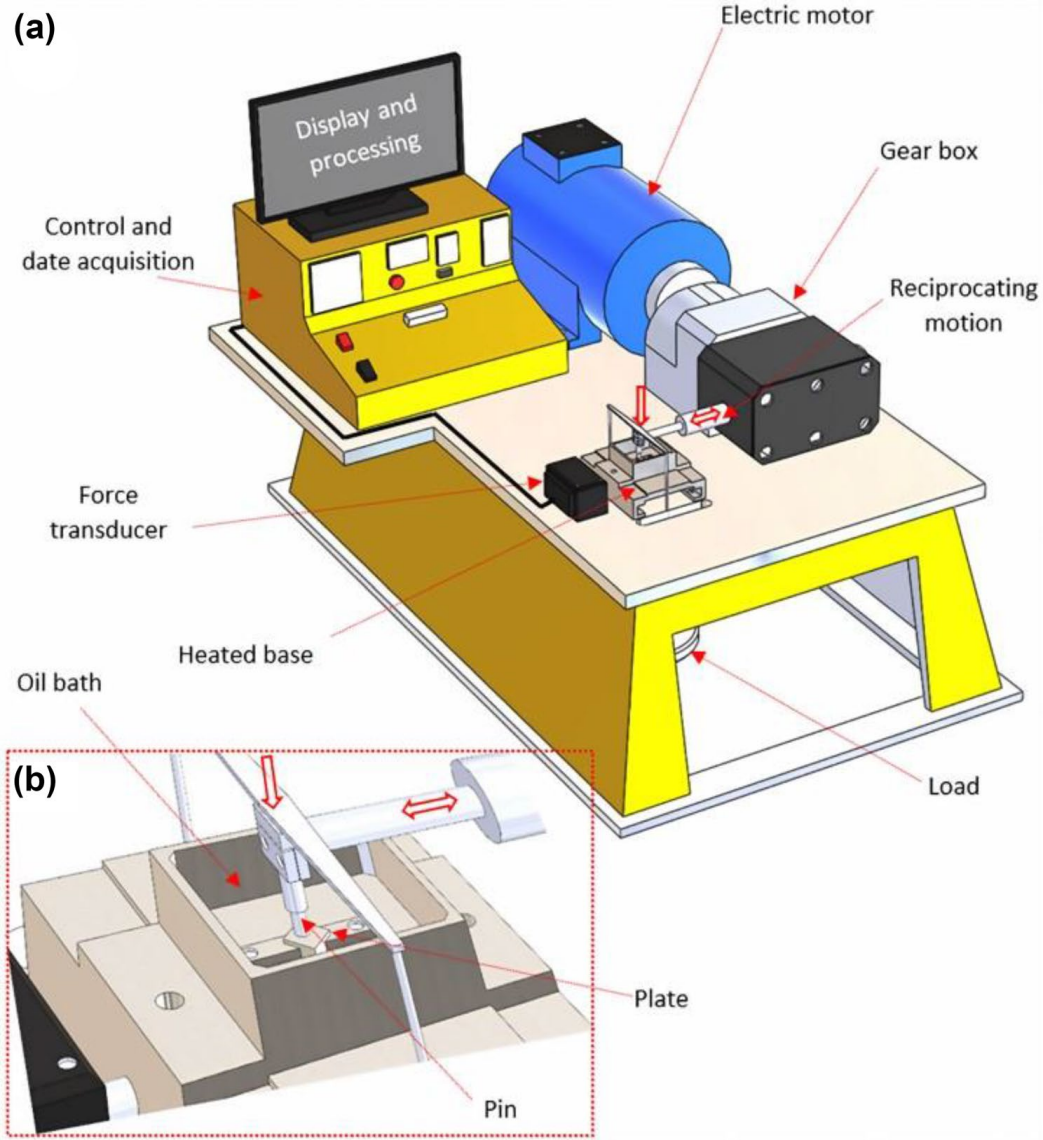
**a** 3D schematic of tribometer (TE77) used in this study, **b** the contact interface between the pin and plate [14]

TE77 tribometer has been used to simulate reciprocating sliding contacts in the engine according to ASTM G 181 [40] as shown in Fig. 5. Table 3 shows the test conditions used in the experiments to investigate the tribological performance of tested oils in boundary lubrication regime (Lambda of fresh oil is λ = 0.27 < 1) [14]. The specification of the pin and plate is described in Table 3. The pin is normally loaded against the stationary plate. The electric motor provides the reciprocating movement through the gearbox. The friction force is measured using the force transducer. The oil temperature is kept at 100 °C to replicate the real working condition in the engine. Tribological tests were repeated at least twice to ensure the repeatability of tests.

**Table 3:** Materials properties of specimens and TE77 set-up parameters

Material properties	Pin	Plate	TE77 parameters	Value
Material	Steel EN31	Steel EN31	Temperature (°C)	100
Dimensions (mm)	10 radius	7 × 7 × 3	Contact pressure (GPa)	1.26
Hardness (HRC)	58–62	58–62	Load (N)	22.1
Roughness (nm)	30–50	400–600	Frequency (Hz)	25
Elastic modulus (GPa)	190–200	190–210	Test duration (min)	120
Poisson’s ratio	0.28	0.28		

An optical white light interferometer (NPFLEX) was used to measure wear on pins after the tribological test. The size of the scar on pin was determined by Vision64 software after analyzing the scanned images. Wear volume loss (spherical cap volume) on pin is calculated using the Eqs. (1) and (2) as follows:

1 $${V}_{pin volume loss}=\frac{\pi .{h}^{2}.(3R-h)}{3}$$

2 $$h=R-(({R}^{2}-{r}^{2}){)}^{0.5}$$

where

$R:$ sphere radius (µm), $h:$ spherical cap height (µm), $r:$ wear scar radius (µm).

Scanning electron microscopy (SEM) technique was used to analyze the surface of wear scar by providing detailed high-resolution images. Energy-dispersive X-ray (EDX) analyser was applied in this study to identify the chemical compositional of tribofilm.

## Results and Discussion

### Soot Removal Effect on Used Oil Performance

**Fig. 6:**
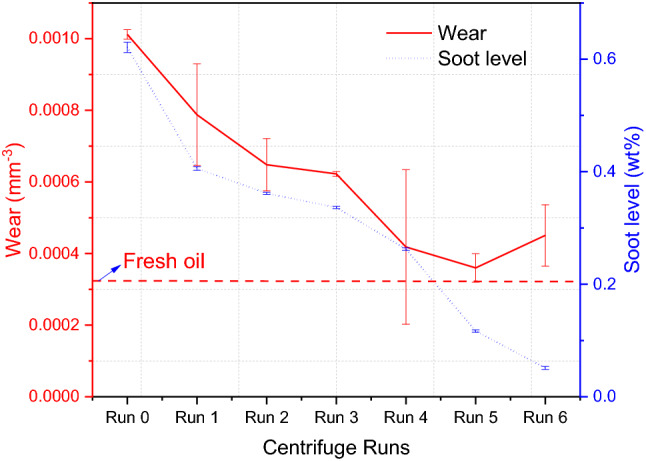
The effect of repeating the centrifuge process on both soot level and wear

Soot in real engine oil has been investigated in serval previous studies [4–8] to determine its effects on wear. The results showed an increase in wear as the soot level increased in the oil. Conversely, removing soot from used oil reduced the amount of wear. Figure 6 shows a decrease in soot level by repeating the centrifuge process. The results reveal a decrease in the wear value with a decrease in the soot level in the used oil with no further decrease in wear after 4-centrifuge runs as displayed in Fig. 6. It is worth noting that wear after removing soot is still higher than the wear of fresh oil sample. This can be due to the additive adsorption on soot and/or antiwear decomposition as FTIR results shown in Fig. 2a. As there was no change in wear values after 4-centrifuge runs, thus the used oil after 4-centrifuge runs is used for further additive replenishment tests. Soot level in used oil was 0.62 wt% after draining the oil from the truck and reduced to 0.26 wt% after 4-centrifuge runs. The results are in line with study [4] confirmed the existence of (< 0.2 wt%) soot in engine oil does not have a significant effect on wear value. While other studies [41–43] found that removing the soot from used oil reduced its effects on wear and improve the oil function.

### Additive Depletion in Used Oil

**Fig. 7:**
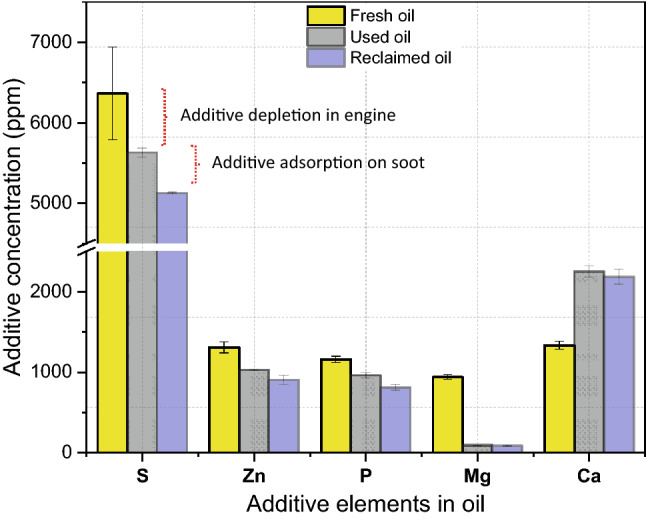
ICP chemical analysis of fresh oil, used oil, and reclaimed oil (after removing soot)

ICP chemical analysis was conducted for used engine oil (0.62 wt% soot) and reclaimed oil after removing soot. Figure 7 indicates the change in the concentration of oil additive after being used in the engine as expected. The results show a decrease in the additive concentration of Zn, P, S, and Mg except Ca concentration. Zn, P, and S elements come from the antiwear additive, such as zinc dialkyl dithiophosphate, and it is expected to be consumed to protect the tribological surfaces. P and S elements could also come from dispersant/detergent compounds such as sulfonates and phosphonates [44]. The main function of detergent additive is to clean and neutralize oil impurities [44]. Ca and Mg elements originate from the detergent compounds. Figure 7 shows an increase in Ca level after being used in the truck. This could come from impurities, rainwater, fuel, or road dust. There is a significant drop in Mg concentration that may be used to overcome impurities and other contaminants that caused an increase in the Ca level.

Additives adsorption on soot particles has been studied before [3, 11–13]. Thus, there is a possibility of removing the additives that are adsorbed on the soot particles during the centrifugation process [45]. Additive adsorption on soot was observed in this study after removing soot as shown in Fig. 7. In general, there was a decrease in the concentration of all elements that originated from both antiwear and dispersant/detergent. The results agree with several studies [3, 11–13] that investigated additives adsorption on soot. However, the effect of additive depletion and additive adsorption on oil performance has not been investigated in previous studies [3, 11–13].

### ZDDP Replenishment of Used Oil

#### Chemical Analysis of Oils

**Fig. 8:**
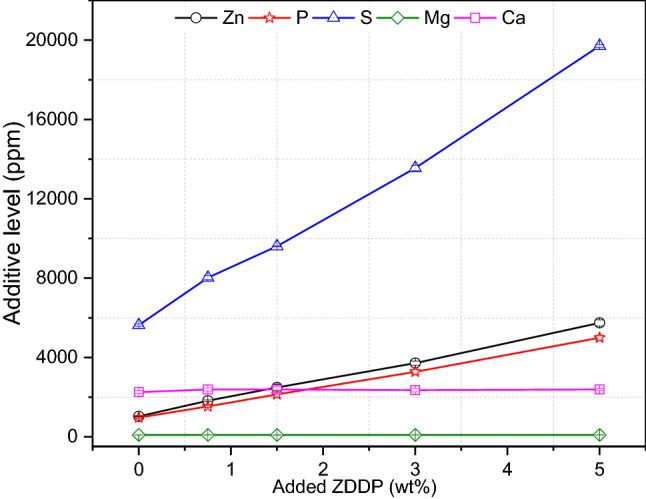
The concentration of additive elements in used oil after adding different levels of ZDDP

Used engine oil was drained after approximately 40,000 km of driving as shown in Table 2. ICP analysis of used oil showed a decrease in most additive elements concentration due to additives depletion as shown in Fig. 7. ZDDP was used in this study to replenish the consumed additive in the used oil and improve its tribological performance. As known ZDDP contains three main elements Zn, S, and P, adding ZDDP to used oil will increase the concentration of these elements. ICP analysis after adding different levels of ZDDP was conducted as shown in Fig. 8. The results show an increase in the concentration Zn, S, and P with no change in Ca and Mg. As the level of ZDDP increases in the used oil, the performance of used oil is expected to improve. The reason behind this is that ZDDP promotes the formation of antiwear tribofilm to protect the rubbing surfaces from oil contamination.

#### Tribological Performance

**Fig. 9:**
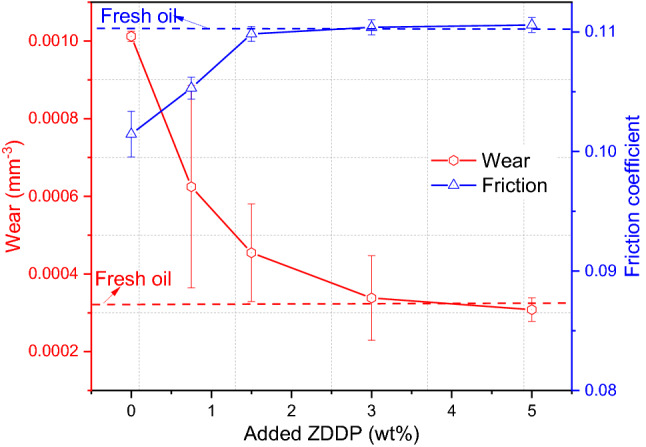
Wear and friction of used oil after adding different levels of ZDDP

Tribological tests of used oil before and after adding different levels of ZDDP have been conducted as shown in Fig. 9. In this study, there is the synergy of two main mechanisms that influence wear and affects the oil functionality after being used in the truck. Firstly, additive depletion or decomposition of antiwear additive that consumed or decomposed in used oil over time causes an increase in wear as illustrated in Fig. 1. Secondly, the existence of 0.62 wt% soot influences tribological performance and causes hard abrasive wear on the rubbing surfaces. After replenishing depleted additives in the used oil, the results demonstrate a consistent decrease in wear with the increase in the level of ZDDP in used oil. Figure 9 reveals that wear of used oil after adding 3 wt% ZDDP performs almost similar to the fresh oil. In this study, it can be concluded that replenishing used oil by adding 3 wt% ZDDP or more was sufficient to renew the oil functionality even in the existence of 0.62 wt% soot.

On the other hand, the soot effect on friction is variable depending on the soot level in the oil [9, 10, 46]. The higher level of soot can cause oil starvation and an increase in friction coefficient [9, 10]. While it has been observed a decrease in friction coefficient with low soot level as soot particles act as friction modifier [46]. In this study, the friction coefficient of used oil contains 0.62 wt% soot decreases before adding ZDDP as shown in Fig. 9. The results in line with studies [46, 47] demonstrated the effect of soot on friction in the existence of soot at low level. Soot acts as a friction modifier causing a decrease in friction coefficient. As ZDDP is added in the oil, a higher friction coefficient resulted [30, 34]. The higher friction coefficient after adding ZDDP can be explained due to the formation of tribofilm derived from ZDDP. The reason behind this is the higher shear strength of tribofilm [34] due to an effective roughening of rubbing surfaces by the formation of uneven distribution of asperity peaks [30]. The effect of shear strength of tribofilm on friction coefficient was less when 1.5 wt% ZDDP or more existed in the used oil as shown in Fig. 9. It appears that tribofilm roughness had a limiting effect on friction with a ZDDP level of ≥ 1.5 wt%.

#### Surface Analysis

**Fig. 10:**
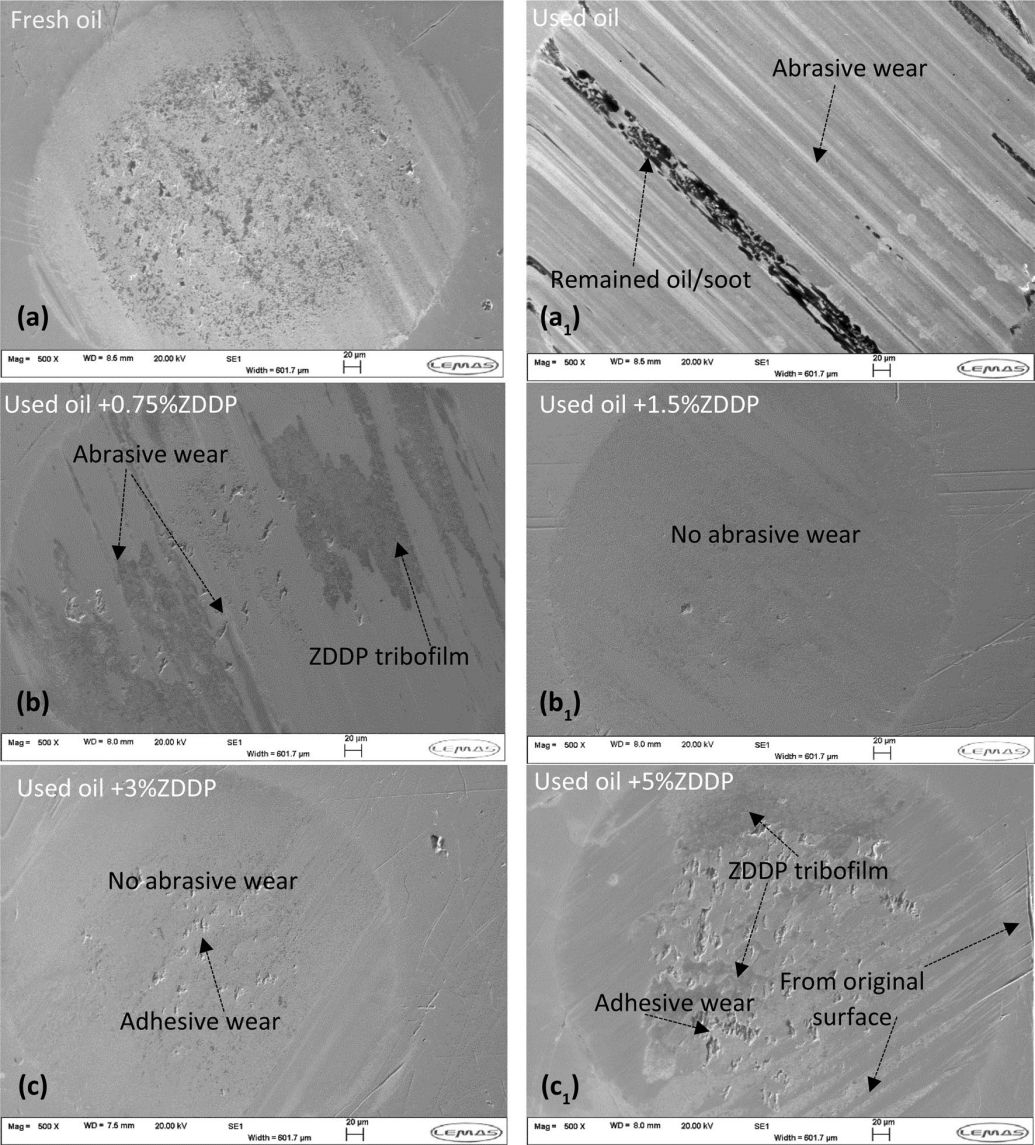
SEM surface analysis of wear scar on pins **a** fresh oil **a**_**1**_ used oil **b** used oil + 0.75wt% ZDDP b_1_) used oil + 1.5wt% ZDDP **c** used oil + 3wt% ZDDP **c**_**1**_ used oil + 5wt% ZDDP

Previous studies [9, 10] revealed that soot in engine oil causes abrasive wear on rubbing surfaces. Figure 10 shows SEM images of wear scar on pins of used oil and after adding ZDDP at different levels. It is expected to see abrasive wear on the surface of used oil in the existence of soot as seen in Fig. 10a1. Adding ZDDP to the used oil reduces the effect of soot on the surface due to the formation of the tribofilm. As known that the ZDDP level increases in the oil, a higher rate of tribofilm growth is expected and this could help to protect the surfaces from soot particles and improve the oil functionality. It is shown in Fig. 10b that adding 0.75 wt% ZDDP to the used oil reduces the effect of soot on surfaces significantly. There is still abrasive wear on the surface as soot overcomes the tribofilm formation and abrades on the surface as shown in Fig. 10b. A higher rate of reforming tribofilm after being removed by soot occurred with a higher level of ZDDP (≥ 1.5 wt% ZDDP). This can protect the surfaces from abrasive wear caused by soot as shown in Fig. 10b1, c and c_1_. It is observed that no abrasive wear was detected on surfaces with uneven distribution of tribofilm as shown in Fig. 10c1. Adhesive wear on surfaces after adding ZDDP has been noted as shown in Fig. 10c, c1. This could be due to the higher shear strength of ZDDP tribofilm between the rubbing surfaces causing adhesive wear [32, 48].

**Fig. 11:**
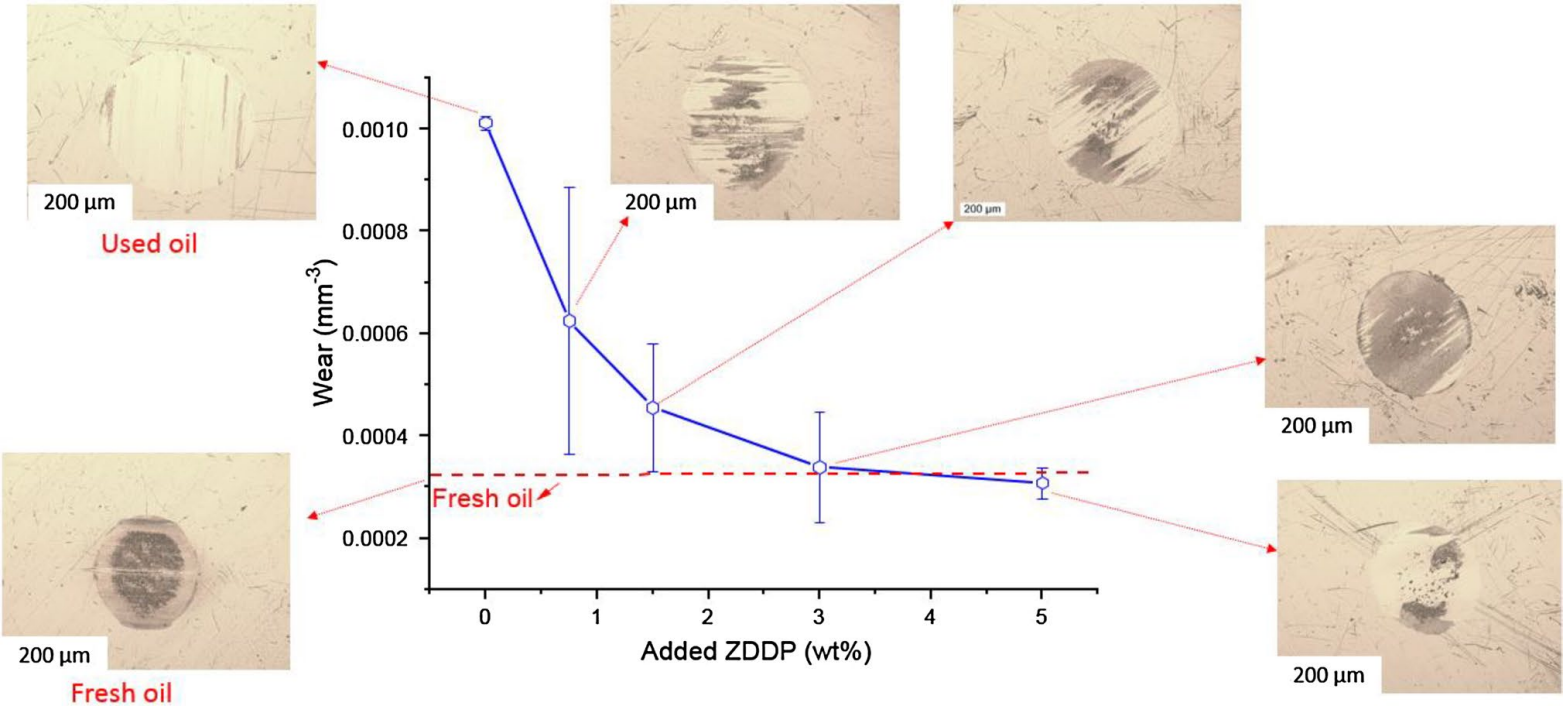
Microscope images of wear scar on pins after adding different levels of ZDDP

**Fig. 12:**
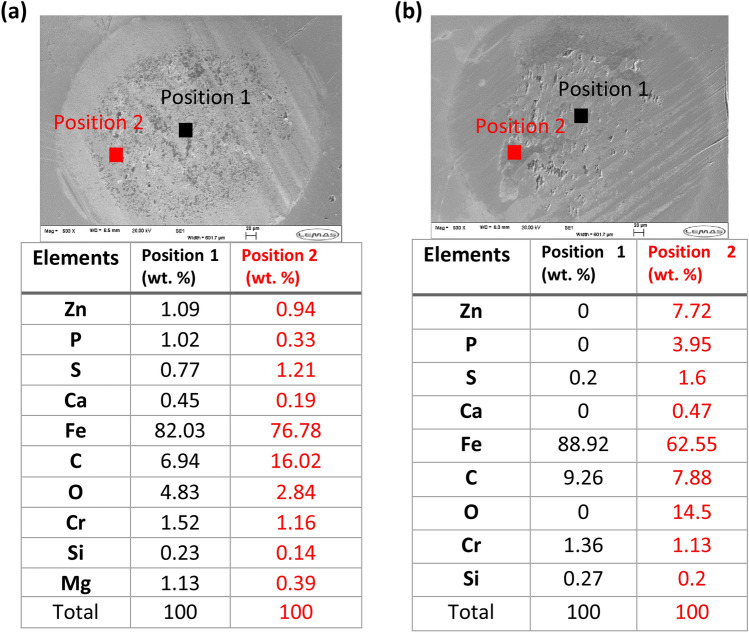
EDX chemical composition of tribofilm of wear scar on pins **a** fresh oil **b** used oil + 5wt%ZDDP samples at two positions. The probed depth in EDX analysis is around 1–3 µm compared to tribofilm thickness which is mostly thinner than 200 nm, which leads to much lower concentrations of P, S, and Zn compared to Fe

Microscope images confirm the distribution of tribofilm after adding ZDDP to the used oil compared to the fresh oil as shown in Fig. 11. Where some regions are covered by tribofilm as shown in a dark area in wear scar, while other tribofilm regions are removed by soot. It is worth noting that ZDDP tribofilm attempts to protect the surfaces from soot particles, but soot abrades and removes the tribofilm from wear scar in some regions. Further chemical analysis of the dark area to confirm the presence of tribofilm was carried out as demonstrated in Fig. 12b. The results prove that the dark area on the surface represents the existence of tribofilm elements on the surface compared to the light area as shown in Fig. 12b.

#### Chemical Composition of Tribofilm

Energy-dispersive X-ray (EDX) analysis was conducted on the wear scar of pins to analyze the chemical composition of tribofilm. Chemical composition of the fresh oil sample at different positions demonstrates the uniform distribution of tribofilm as displayed in Fig. 12a. EDX results revealed full removal of the whole tribofilm causing abrasive wear on the surface in the existence of soot as shown in Fig. 10a1. Adding ZDDP increased the concentration of three main elements S, Zn, and P in the used oil as shown in Fig. 7. Therefore, the concentration of these elements in the tribofilm composition is also increased compared to fresh oil as expected (Fig. 12b at position 2). Tribofilm was not uniform on the surface after adding ZDDP (Fig. 11) and this can be confirmed by chemical analysis of tribofilm at different positions. The results proved the distribution of tribofilm elements in wear scar after adding ZDDP and in the presence of soot is uneven (Fig. 12b). The chemical composition of tribofilm reveals the coverage of the tribofilm at some regions and the removal of tribofilm by soot at other regions. The results conclude that there is no full coverage of tribofilm on the surface after adding ZDDP even at a high level (5wt%) in the existence of soot particles. Soot can overcome the formation of tribofilm and remove the tribofilm in most surface regions.

### ZDDP Replenishment of Reclaimed Engine Oil

#### Tribological Performance

**Fig. 13:**
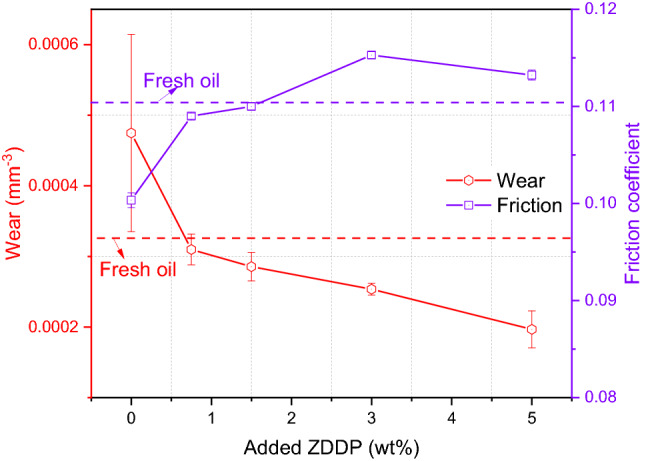
Wear and friction of reclaimed after adding different levels of ZDDP

Used oil after removing soot performed higher wear compared to fresh oil due to the decomposition of antiwear additive and additive depletion as displayed in Figs. 1 and 6, respectively. Adding ZDDP at different levels to the reclaimed oil had a positive effect and decreased the amount of wear significantly as shown in Fig. 13. As the ZDDP level increased in the oil, the amount of wear decreased gradually and the replenished oil performed better than fresh oil after adding 0.75 wt% ZDDP. Replenishing the reclaimed oil with a level ≥ 0.75 wt% ZDDP is sufficient to improve wear and extend the oil function. Further surface investigations are discussed in the next section to ensure no abrasive wear and fully tribofilm coverage on the surface after replenishing the additives.

Friction results as shown in Fig. 13 reveal that the friction coefficient of reclaimed oil was less than the friction coefficient of fresh oil due to the presence of 0.26 wt% soot. Existence of these particles in oils acted as a friction modifier between rubbing surfaces reducing the friction. However, it is good to mention that the existence of 0.26 wt% soot did not affect wear value as shown in Fig. 6. Friction increased with an increase in the amount of ZDDP in reclaimed oil as shown in Fig. 13. Friction coefficient starts leveling with the amount of ≥ 3 wt% ZDDP. The increase in friction coefficient after adding ZDDP to the reclaimed oil is due to the high roughness of tribofilm [30] or the higher shear strength of tribofilm [34]. The effect of the increase in ZDDP level on the coefficient of friction at the higher level is limited. The results are agreed with studies [32, 48] that demonstrated the effect of tribofilm on the friction coefficient can be stabilized at the high level of ZDDP.

#### Surface Analysis

**Fig. 14:**
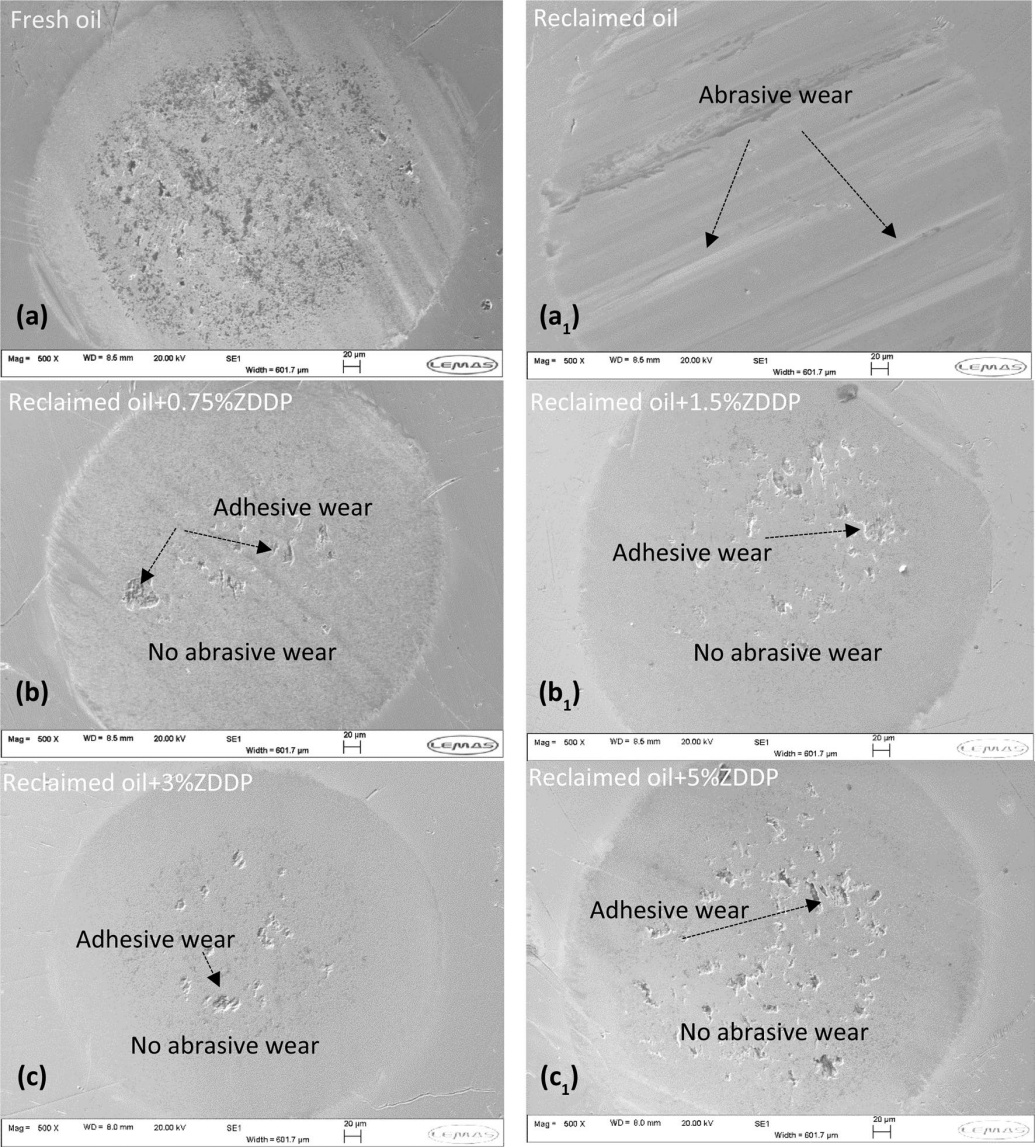
SEM surface analysis of wear scar on pins **a** fresh oil **a**_**1**_ reclaimed oil **b** reclaimed oil + 0.75wt% ZDDP **b**_**1**_ reclaimed oil + 1.5wt% ZDDP **c** reclaimed oil + 3wt% ZDDP **c**_**1**_ reclaimed oil + 5wt% ZDDP

Figure 14 shows the wear scar of pins after replenishing the reclaimed oil at different levels of ZDDP compared to reclaimed and fresh oil samples. Abrasive wear was detected on the wear scar surface of reclaimed oil (Fig. 14a1) due to depletion and decomposition of additives. It was found that wear value reduced significantly after adding 0.75 wt% ZDDP and performed better than fresh oil as shown in Fig. 13. The surface of wear scar after adding 0.75 wt% ZDDP showed no abrasive wear and full coverage of tribofilm as shown in Fig. 14b. Additive replenishment at higher ZDDP percentage 1.5, 3, and 5 wt% revealed no abrasive wear and uniform tribofilm formation as shown in Fig. 14b1,c, and c_1_. Adhesive wear is observed after replenishing the reclaimed oil as displayed in Fig. 14b, b1, c, and c_1._ This mostly comes from the higher shear strength of ZDDP tribofilm between the rubbing surfaces causing adhesive wear [32, 48].

**Fig. 15:**
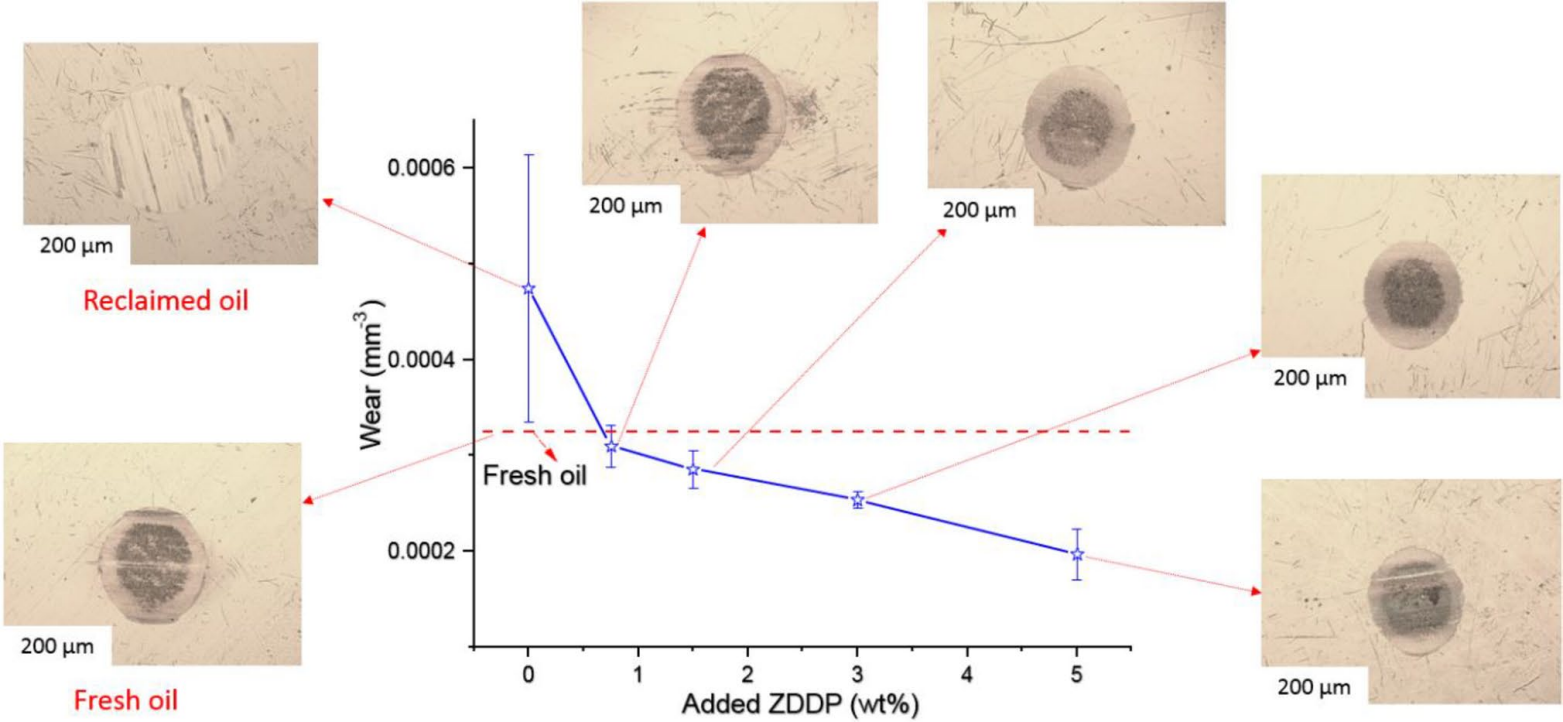
Microscope images of wear scar on pins after adding different levels of ZDDP

Microscope images as shown in Fig. 15 display the distribution of tribofilm after adding ZDDP to the reclaimed oil compared to fresh oil. Where all regions on wear scar are protected by tribofilm represented as a dark area on wear scar. It is evident that no abrasive wear and full coverage of ZDDP tribofilm after replenishing the reclaimed oil.

#### Chemical Composition of Tribofilm

**Fig. 16:**
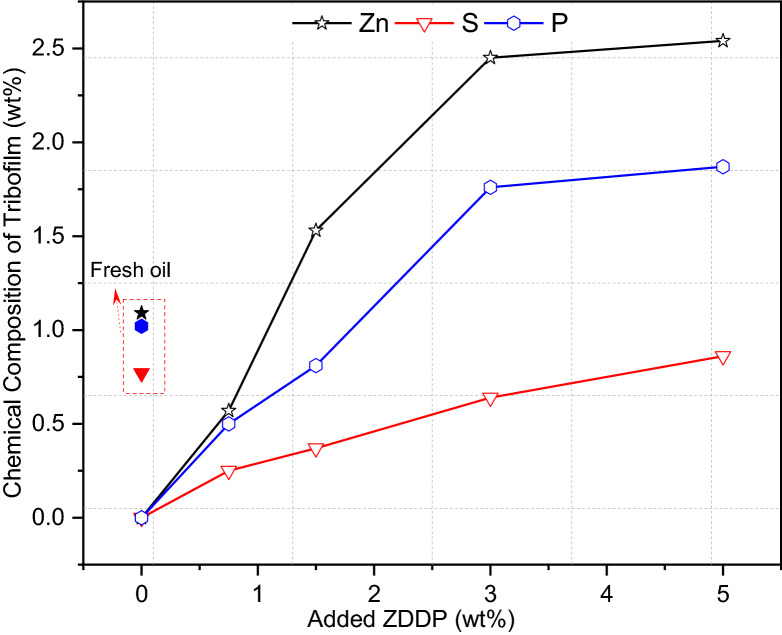
EDX chemical composition of tribofilm of wear scar on pins after adding ZDDP to the reclaimed oil

**Fig. 17:**
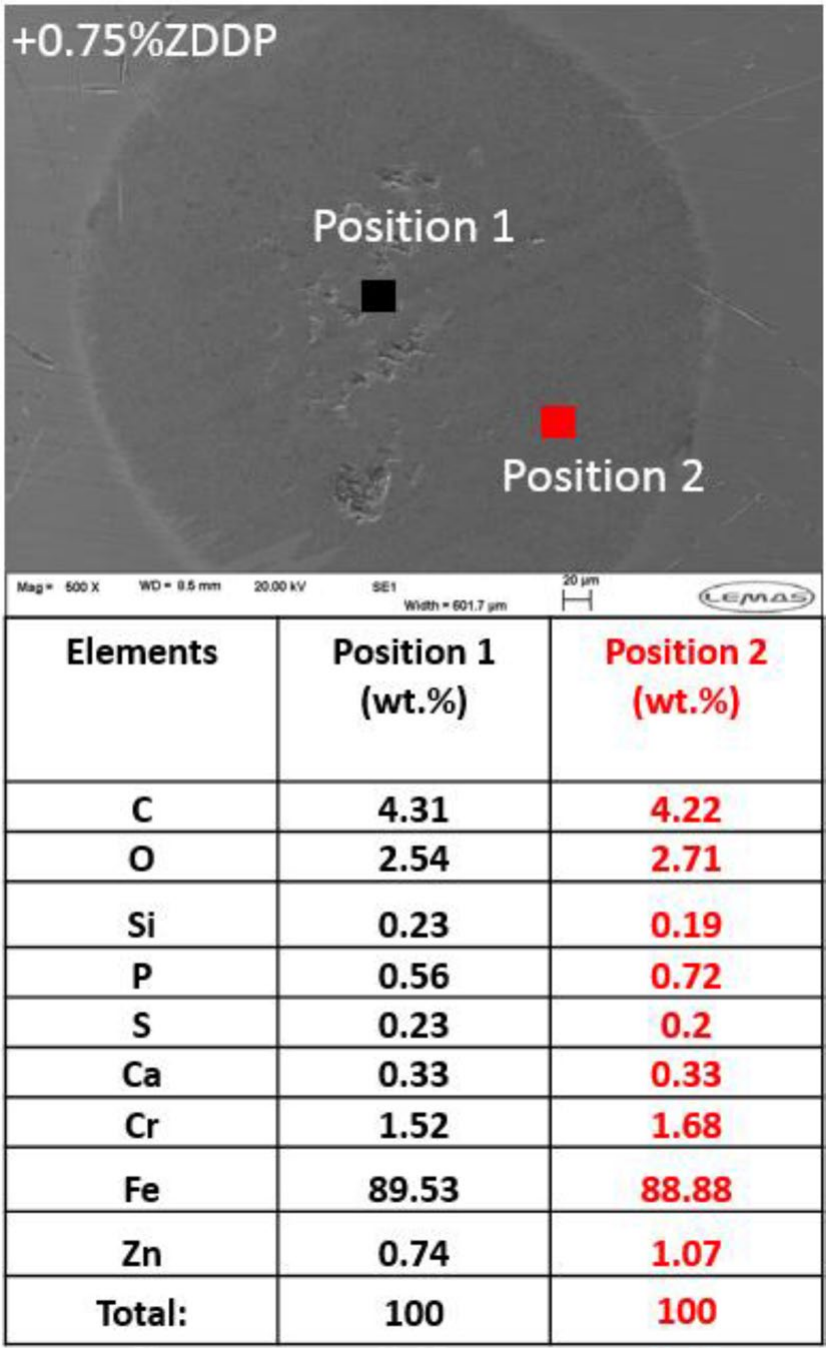
EDX chemical composition of tribofilm on the pin for the reclaimed oil + 0.75wt% ZDDP sample at two positions

EDX analysis of wear scar of pins after adding ZDDP at different levels to the reclaimed oil is presented in Fig. 16. The results obtained from the chemical analysis of tribofilm of the reclaimed oil sample found no ZDDP tribofilm on the surface (Fig. 16). The results of the chemical analysis of tribofilm after adding ZDDP to the reclaimed oil confirms the existence of tribofilm on the surface. The reduction in the amount of wear after adding ZDDP (Fig. 13) is due to an increase in the thickness of ZDDP tribofilm. The presence of ZDDP in the reclaimed oil influences the chemical concentration of main tribofilm elements such as S, Zn, and P. Figure 16 revealed that the higher level of ZDDP in the reclaimed oil was, the higher concentration of ZDDP elements was found in tribofilm. The tribofilm distribution on the surface after adding ZDDP to reclaimed oil was uniform as shown in Fig. 17. Figure 17 demonstrates that the chemical analysis of tribofilm at different positions approximately has similar chemical concentrations of Zn, S, and P. This leads to a conclusion that replenishing the reclaimed oil can improve the tribological performance and protect the surface by forming uniform tribofilm similar to the fresh oil.

## Conclusions

This study investigates the effect of ZDDP replenishment on the tribological performance in heavy-duty used oil. It was observed that the chemical structure of bulk oil did not change overuse in the heavy-duty engine. However, the performance of used oil was influenced by three main mechanisms such as the existence of soot, additive depletion, and the decomposition of antiwear additive. ZDDP replenishment at different levels has been investigated for used oil containing soot and after removing soot. The main conclusions from this study can be summarized as follows:

- Wear decreases gradually with the reduction in soot percentage in the oil until 0.26 wt% and then there was no change in wear value coming from the soot.
- Chemical analysis of heavy-duty used oil using FTIR demonstrates no change in the chemical structure of the oil, but decomposition of antiwear additive occurred.
- The reduction in the elemental concentration of additives is due to additive depletion in the engine overuse and additive adsorption on soot after being removed.
- Antiwear additives decomposition, additive depletion, and additive adsorption on soot cause an increase in wear and abrasive wear on the surfaces.
- ZDDP replenishment process of used oil shows a higher level of ZDDP (3 wt%) is required to improve wear value to be similar to fresh oil. Microscope and SEM images of wear scar reveal no abrasive wear on the surface after adding a high level of ZDDP. While EDX results show uneven distribution of tribofilm on the surface in the existence of soot.
- ZDDP replenishment of the reclaimed oil demonstrates less amount of ZDDP (0.75 wt%) is required to improve wear value to be similar to fresh oil.
- Surface analysis of reclaimed oil after adding 0.75 wt% ZDDP shows uniform distribution of tribofilm and no abrasive wear was detected on the surface.
